# Exploring the Anatomical Basis of Effective Connectivity Models with DTI-Based Fiber Tractography

**DOI:** 10.1155/2008/423192

**Published:** 2008-05-05

**Authors:** Hubert M. J. Fonteijn, David G. Norris, Frans A. J. Verstraten

**Affiliations:** ^1^Helmholtz Institute, Universiteit Utrecht, Heidelberglaan 2, 3584 CS Utrecht, The Netherlands; ^2^F.C. Donders Centre for Cognitive Neuroimaging, Radboud University Nijmegen, P.O. Box 9102, 6500 HC Nijmegen, The Netherlands

## Abstract

Diffusion tensor imaging (DTI) is considered to be a promising tool for revealing the anatomical basis of functional networks. In this study, we investigate the potential of DTI to provide the anatomical basis of paths that are used in studies of effective connectivity, using structural equation modeling. We have taken regions of interest from eight previously published studies, and examined the connectivity as defined by DTI-based fiber tractography between these regions. The resulting fiber tracts were then compared with the paths proposed in the original studies. For a substantial number of connections, we found fiber tracts that corresponded to the proposed paths. More importantly, we have also identified a number of cases in which tractography suggested direct connections which were not included in the original analyses. We therefore conclude that DTI-based fiber tractography can be a valuable tool to study the anatomical basis of functional networks.

## 1. INTRODUCTION

In functional neuroimaging, and particularly in PET and fMRI, study design and analysis have been dominated by the concept of functional *segregation*, which emphasizes the specialization of a brain structure for a specific part of a cognitive function. This has resulted in a large number of studies in which differences in cognitive states are linked to the differential activation of separate brain areas [1]. The concept of functional *integration*, on the other hand, has long been recognized as an equally important principle of brain organization. Functional integration refers to the interaction between brain areas and has been studied with two categories of analyses: functional connectivity analyses and effective connectivity analyses. In functional connectivity analyses [2–4], the covariance structure of a measure of brain activity is studied, from which differences in cognitive states are linked to differences in correlations between regions. These analysis methods are thus limited in their capacity to make inferences about the directionality of these correlations, which makes it difficult to address, for instance, the functional hierarchy of the brain structures under investigation. In effective connectivity analyses, on the other hand, models are defined a priori, comprising the brain structures of interest and assumptions about the afferent/efferent connections between them [5, 6]. These models are then fitted to the activity of these brain areas to obtain the strength of these connections, which enables inferences on changes in connection strengths in relationship to cognitive states.

Effective connectivity has been defined as the influence one neural system or region exerts over another [7]. There are two ways by which this influence can be mediated: via a direct path between two regions, or via an indirect path in which a third region is involved. The analysis methods that have been applied to functional neuroimaging data, structural equation modeling (SEM) and dynamic causal modeling (DCM), differentiate between these two possibilities, provided that the underlying model (regions and their paths) is completely specified. If two regions are connected via a third region and this third region has furthermore no other influence on the rest of the network, one can choose to incorporate only an indirect path between the two regions and to refrain from explicitly modeling the third region and its direct paths. This is usually done to keep the model computationally tractable. We are working here, however, on the premise that most of the specified paths should reflect veridical direct paths, because we believe that too many “indirect” paths, involving these “third party” regions, which are not explicitly taken into account in the model, will seriously decrease the biological validity of the model.

SEM has also extensively been applied in the social sciences, where these paths represent abstract causal connections between variables. When this method is applied to functional neuroimaging however, these paths should ultimately correspond to the white matter connectivity of the brain regions under investigation. This hence yields an extra source of model validation. Furthermore, McIntosh and Gonzalez-Lima [8] have studied the effect of erroneous model specification on the estimation of the path coefficients and have found that it could seriously impinge on the estimation of these coefficients. Therefore, it would be highly valuable to incorporate all available knowledge regarding anatomical connections into effective connectivity models. It should be noted that we do not imply that each existing anatomical connection should result in an effective connection in each and every cognitive task. However, it is the role of the model estimation to indicate which anatomical connections have become effective for which task manipulation. Therefore, leaving an anatomical connection, which is known to exist, out of a model specification can only be justified when one has strong beliefs about the functioning of this path in the present context, possibly combined with a requirement for a decrease in model complexity.

Unfortunately, our knowledge about human anatomical connectivity is relatively sparse, because a number of techniques that are used in other species (active tracers) are highly invasive. Therefore, only two classical anatomical methods can be used: *dissection studies*, which only provide information on a relatively coarse scale, and *passive tracer studies*, which are not very well suited to investigate long-range connectivity. Most effective connectivity studies thus can only validate their connections with information from nonhuman primates, which in turn raises problems with the homology of brain structures between different species.

In the last decade, a technique known as diffusion tensor imaging (DTI) [9, 10] has emerged as a good candidate to resolve this situation. In DTI, the sensitivity of the (diffusion-weighted) MR signal to the self-diffusion of water on a microscopic scale is employed to characterize the anisotropic structure of white matter in vivo. It is assumed that the direction in which diffusion is largest is collinear to the direction of the axonal bundle in the voxel, because diffusion is assumed to be hindered in directions perpendicular to this direction. With this information, fiber tracking [11, 12] can be performed in which the main diffusion directions of voxels are followed throughout white matter. This method already has provided useful insights in, for instance, the anatomy of the thalamus and the striatum [13, 14].

So far, researchers have acknowledged a number of limitations to this technique. First, DTI provides no information about the afferent or efferent character of the axons, because the diffusion of water does not differentiate between these two situations. Second, the tensor model can only provide one main direction per voxel, which considerably increases the likelihood of erroneous tracking results through a region of crossing fibers. Finally, in cerebral gray matter there is generally no dominant fiber direction, making it difficult to track fibers to their cortical origin.

The aim of the current study is to investigate to what extent DTI-based tractography can provide support for the anatomical basis of the networks, proposed in effective connectivity studies. Furthermore, we have investigated whether DTI-based tractography is able to reveal any connections that go beyond the ones proposed in the original analysis. To address these questions, we have chosen eight effective connectivity studies all using structural equation modeling. SEM was introduced at an early stage of the development of PET and fMRI, with the consequence that a body of SEM studies is to be found in the literature. DCM, on the other hand, has only relatively recently been introduced, but is gaining a rapid popularity. It should be noted that any conclusions we are able to draw in this study within the context of SEM models can be readily generalized to DCM studies as there is no difference in the role of the underlying anatomical model in both frameworks.

We have chosen networks spanning a number of different cognitive domains, including learning [15, 16], cognitive control [17, 18], working memory [19], visual and auditory perception [20], major depression [21], and the thalamocortical network involved in general-anaesthetic-induced unconsciousness [22]. We performed a standard DTI experiment on 6 subjects and used the coordinates of the network nodes as seed regions for a fiber tracking analysis. In this analysis, we have established whether DTI-based fiber tractography provides evidence about the direct nature of every possible connection, whether or not it was proposed in the original studies.

## 2. MATERIALS AND METHODS

### 2.1. Subjects

We studied 6 healthy subjects (2 females, age range 25–32 years) after informed consent was given according to institutional guidelines of the local ethics committee (CMO protocol region Arnhem-Nijmegen, The Netherlands).

### 2.2. Imaging

DTI was performed using a twice refocused pulsed gradient spin echo EPI sequence [23] at 1.5 T(Sonata system, Siemens, Erlangen, Germany)with a standard head coil. Axial slices were obtained using the following imaging parameters: repetition time = 9900 milliseconds, echo time = 88 milliseconds, flip angle = 90°, 128 × 128 matrix, 320 mm × 320 mm field of view, and slice thickness = 2.5 mm with no gap (2.5 × 2.5 × 2.5 mm isotropic voxels). Diffusion weighting was obtained along sixty noncollinear directions [24] using a *b*-value of 700 s/mm^2^. Five reference images with no diffusion weighting were also obtained. This resulted in a scanning time of approximately 10 minutes per subject.

### 2.3. Analyses

For each subject the five reference images with no diffusion weighting were averaged and normalized to the MNI T2 template in SPM2 (Statistical Parametric Mapping, http://www.fil.ion.ucl.ac.uk/spm). The matrix of normalization parameters was inverted to obtain the transformation matrix from standard space to world space. We have done this to avoid the extensive resampling and reorientation of the data that is involved in the normalization of DTI data [25, 26], because we hypothesize that this would lead to a degradation of the finer details in the fiber tracts.Only linear terms were used in the normalization to ensure that the transformation matrices could be inverted. No motion correction was applied. Diffusion tensors and fractional anisotropy (FA) maps were calculated using the diffusion toolbox [27] in SPM2. The FA maps were used for displaying the anatomical location of the ROI coordinates. These anatomical locations were then used as seed regions for fiber tracking. Fiber tracking was performed in the DTI-Studio package [28] using the FACT algorithm [29]. Tracking was terminated when the angle of two consecutive eigenvectors was larger than 85°, or when a voxel was reached with an FA value smaller than 0.20.

In most of the original studies [15–19, 22], the ROIs that were used for effective connectivity analysis were all spheres of 8 mm radius. However, in two studies [20, 21] only the peak voxels of a partial least-squares analysis [30] were used. Because these voxels represented larger clusters of voxels, we have used ROIs with roughly the same size (spheres of 8 mm radius) as a starting point for all networks. We have chosen not to transform the whole original ROI into subject space, as this might lead to extensive seeding of the white matter adjacent to the seed coordinate, and thus to many false positives. Instead, we have drawn ROIs on the individual subject’s FA maps, taking care that the borders of the ROI were at the border of gray and white matters (as visible on the FA map) and that the ROI would approximately be of the same size as the original ROI. All possible combinations of regions were tracked, including the connections that were not proposed in the original effective connectivity studies.

## 3. RESULTS

**Figure 1:**
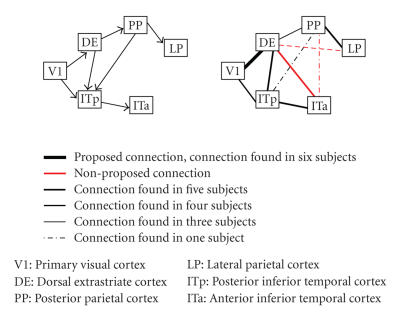
The proposed network of Büchel et al. is shown on the left. The connections found in our analysis are shown on the right. The legend indicates the meaning of the color scale and the thickness of the lines. This legend is valid for all the following figures.

**Figure 2:**
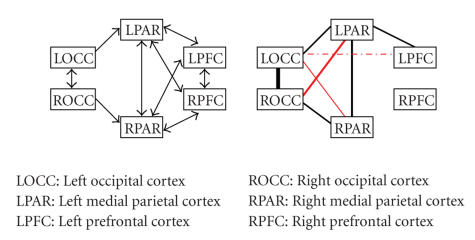
The network and results for Fletcher et al.

**Figure 3:**
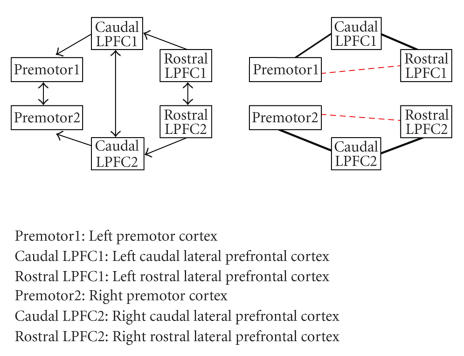
The network and results for Koechlin et al.

**Figure 4:**
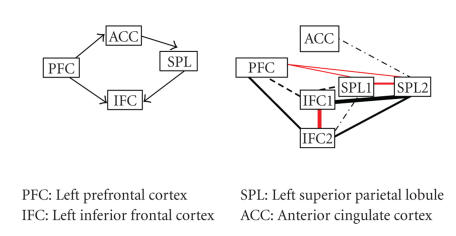
The network and results for Kondo et al.

**Figure 5:**
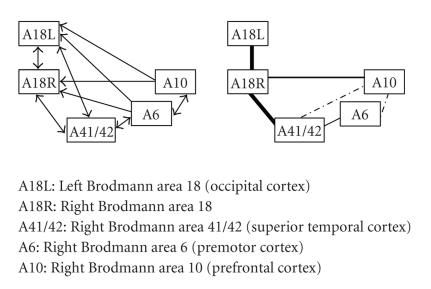
The network and results for McIntosh et al.

**Figure 6:**
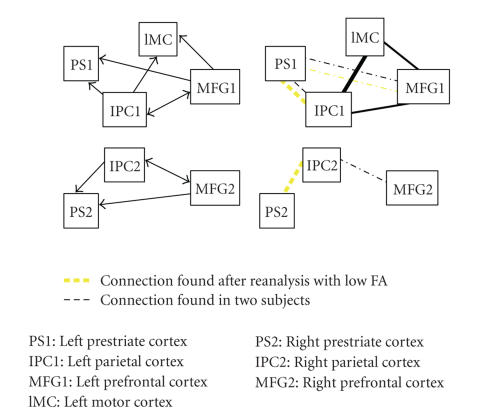
The network and results for Rowe et al.

**Figure 7:**
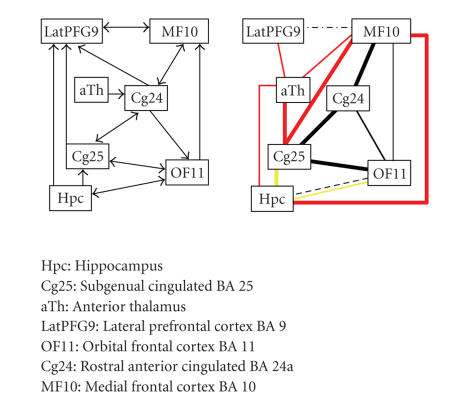
The network and results for Seminowicz et al. BA = Brodmann area.

**Figure 8:**
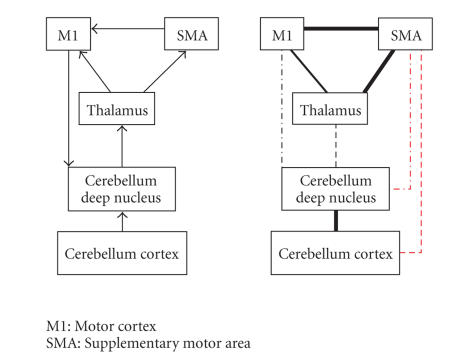
The network and results for White et al.

The results for all networks under investigation are visualized in Figures 1–8, in which the thickness of the connecting lines indicates the number of subjects in which a particular connection was found. In the appendix, we have also listed these results in Table 1. Our results support the proposed paths to a large extent. The most striking class of paths which are not supported by our findings contains frontal interhemispheric paths. This can however convincingly be explained by methodological shortcomings in regions with crossing fibers in the frontal parts of the brain. We will discuss these issues further in Section 4. In half of the studies, we have also found connections indicating paths that were not included in the original studies. In the following, we will describe these findings for each study separately.

The nomenclature of the original studies is maintained throughout this whole article. This not only leads to the situation that this nomenclature is inconsistent between studies, but may also give the incorrect impression that similar connections are under investigation in different networks. It is therefore important to note that the regions of interest normally are spheres of approximately 8 mm in diameter, and thus two regions with the label “prefrontal cortex” can be quite widely separated.

**Büchel et al.**

The network of this study can be separated into a dorsal stream (V1, DE, PP, and LP) and a ventral stream (V1, ITp, and ITa) of visual areas. The paths within these streams are supported in the majority of subjects by our DTI results. However, the crucial path under investigation is the path between the two streams (PP-ITp), which is hypothesized to mediate the learning effect under investigation. Interestingly, we have found no evidence for this path, but we have found support for a path (DE-ITa), which was not included in the proposed network and which could be a potential candidate to mediate this effect. Whereas we do not wish to suggest that the original path (PP-ITp) should be dismissed, it would be interesting to investigate whether a part of the learning related effects, reported in the original study, is mediated by the new path we have reported.

**Fletcher et al.**

In this network, a series of regions (OCC, PAR, and PFC) is proposed in both hemispheres with symmetrical paths within the hemispheres and extensive interhemispheric connections. The paths between occipital and parietal cortices are supported by our findings as are the interhemispheric connections between these regions. Furthermore, we have found evidence for paths that were not included in the original model, namely, interhemispheric connections between occipital and parietal cortices. This is quite remarkable, because interhemispheric connections between nonhomologous regions are rarely found in this kind of analyses.

Also of interest are the cases in which the proposed paths are not supported by our results. The interhemispheric connections we did not find (LPFC-RPFC, LPFC-RPAR, and RPFC-LPAR) fall into the aforementioned problematic class of frontal interhemispheric connections. The most interesting negative result is the lack of connections between right parietal and right prefrontal cortices, in contrast to the presence of these connections in the left hemisphere. In this case, methodological shortcomings are not likely to affect the results, as this would indicate that these shortcomings would exist for the right hemisphere but not for the left hemisphere. The same argument makes the consideration of connections via an extra region, which has not been included in the network, also unattractive. Therefore, we tentatively interpret these findings as a support for an asymmetry in connectivity between these areas in parietal and prefrontal cortices.

**Koechlin et al.**

This network contains a set of motor and prefrontal regions in both hemispheres which are symmetrically connected within the hemispheres. Furthermore, homologous areas are connected with each other. This last set of paths falls again in the class of frontal interhemispheric connections and thus it is not surprising that we have found no support for these paths. We have found connections supporting all intrahemispheric paths in most subjects. Interestingly, Koechlin et al. have proposed an alternative network in their study, which contained also paths directly from premotor cortex to rostral prefrontal cortex. This extra network did not result in significant changes in the original paths, which is in agreement with the lack of support for these paths in our results.

**Kondo et al.**

Kondo et al. have proposed four network nodes (PFC, ACC, SPL, and IFC). However, two of these nodes (SPL and IFC) can be further split up in four anatomically distinct regions (SPL1, SPL2, IFC1, and IFC2). We have thus decided to treat each of these regions as a separate node and have studied the connections between these nodes. Our results show that the regions which constitute each original node (e.g., IFC1 and IFC2) are connected with each other, but they show differential connectivity patterns with the rest of the network, which suggest that they also have a different role within this network. The lack of support for paths from and to the ACC was surprising, especially given the fact that the ACC is known to connect extensively with the prefrontal cortex in the macaque [31].

**McIntosh et al.**

In this network, a set of regions is proposed ranging from occipital, temporal, premotor, and prefrontal regions. Moreover, these regions were proposed to be highly interconnected. Given our results in the other studies, it was not surprising that there was hardly any support for the (nonhomologous) interhemispheric connections originating in frontal and prefrontal cortices. Support was found for paths between occipital cortex and frontal and superior temporal cortices. However, there are also two intrahemispheric paths for which no support was given (A6-A18R, A10-A6). Furthermore, there was no evidence found for paths which were not included in the original analysis. Therefore, within the context of this network, DTI did not deliver any extra information.

**Rowe et al.**

Rowe et al. have proposed a network with bilateral parietal, prefrontal, and prestriate areas and a motor area in the left hemisphere only. We have found only scarce support for the paths to and from prestriate area, even after lowering the FA threshold. In the left hemisphere, we have found consistent evidence for all other proposed paths. However, as in the network of Fletcher et al., we have found support for parietal-frontal connections in the left but not in the right hemisphere. Based on the same arguments as in the network of Rowe et al., we again interpret this as an evidence for an asymmetry in these connections. This is even more remarkable, because the locations of parietal and especially prefrontal areas in both studies are quite widely separated.

**Seminowicz et al.**

The network of Seminowicz et al. consists predominantly of prefrontal subcortical areas in the right hemisphere, with the lateral prefrontal cortex as the only region in the left hemisphere. This might directly explain why we have found only scarce evidence for paths from this region to the rest of the network, because of the aforementioned methodological problems with frontal interhemispheric connections.

In contrast to the study of Kondo et al., we have found connections supporting the proposed paths from the anterior cingulate cortex. Furthermore, we have found many connections from the thalamus to the rest of the network which do not conform to proposed paths. This is however not surprising, as it is well known that the different nuclei in the thalamus extensively connect to different parts of the cortex [13]. The “extra” connections are thus most probably the result of the fact that the seed region in this case was too large to specifically select the nucleus of the thalamus involved in this network. Further evidence for paths that were not proposed in the original network was found for connections from medial prefrontal cortex to the hippocampus and the subgenual cingulated cortex.

The hippocampus is a region of low FA and thus in an initial analysis showed only very limited connectivity with the rest of the network. We have therefore repeated the analysis with a lowered FA threshold (0.15) which yielded support for the proposed paths from the hippocampus.

**White et al.**

White et al. have proposed a model with the left motor cortex and supplementary motor area, the thalamus, and two areas from the right cerebellum. We have found support for the intrahemispheric paths but not for the interhemispheric ones. This last finding contradicts findings from the macaque literature in which there are connections found between the primary motor cortex and the contralateral cerebellum deep nucleus [32]. Moreover, there is evidence that the pons, which is a region through which these fibers have to pass, contains crossing fibers [33]. We, therefore, conclude that it is valid to include these connections in an effective connectivity analysis.

## 4. DISCUSSION

In this study, we have for the first time used DTI-based fiber tractography to investigate the anatomical basis of effective connectivity models. The aim of this study was twofold. First, we wanted to establish that DTI-based tractography is able to resolve the connectivity between ROIs of the size typically used in effective connectivity studies. We hypothesized that the majority of the proposed paths were indeed valid, and compared the results of our DTI-based analysis with these paths. We have found positive evidence for a substantial number of paths. The negative findings will be discussed below. However, we believe that the greatest potential advantage of using DTI-based tractography in the context of effective connectivity models lies in establishing paths that are not suggested by the available knowledge (e.g., macaque tracer literature). Our second aim was therefore to investigate the evidence for connections which were not proposed in the original studies. We have found evidence for such connections in half of the models. In the following, we will discuss these findings in the context of current methodological limitations and we will evaluate their implications for the proposed models.

When there was no evidence found for a proposed path, there are two explanations possible:

- there is no direct anatomical connection; in this case, the proposed path can only be supported by an indirect connection with a third region;
- there is a direct connection, but it has not been found due to methodological limitations (false negative).

The second explanation is especially relevant to a class of frontal interhemispheric connections. As mentioned in the introduction, DTI-based tractography has profound difficulties when tracking through voxels with multiple fiber populations. The frontal interhemispheric connections pass through the corona radiata, which is well known to contain such voxels [34]. Therefore, any negative findings about this class of connections have to be interpreted as being inconclusive.

There is another set of negative findings which merits further discussion: in both networks of Fletcher et al. and of Rowe et al., we have found evidence for a connection between parietal and prefrontal cortices in the left hemisphere but not in the right hemisphere Here, an explanation in terms of methodological limitations seems unattractive, because this would suggest that these limitations occur in the right hemisphere but not in the left hemisphere. Therefore, we tentatively conclude that these connections are indeed absent in the right hemisphere and that this is an evidence for an asymmetry in parietal-frontal connectivity. We should point out, however, that this asymmetry seems counterintuitive at first, since the right hemisphere is hypothesized to be dominant for visuospatial processing and one would expect that these connections would be especially strong for this hemisphere. However, as we are dealing with relatively small ROIs within the frontal and parietal lobes, this does not rule out the possibility of there being any connections between these lobes in the right hemisphere.

There are a number of paths for which no evidence was found and which cannot be interpreted directly in terms of methodological limitations. As in many neuroimaging studies, these negative findings are difficult to classify and it would certainly be imprudent to conclude that these paths do not exist. Moreover, it is also possible that a path is mediated by connections from and to a third region, which was not included in the network. This situation can be relatively harmless if the function of the missing region is known to be restricted to “message passing”. However, we hypothesize that, if there is a substantial number of such “indirect” connections present in a network, the inclusion of these other regions, which mediate these connections, becomes necessary to ensure the biological validity of the network.

Given the methodological issues discussed above, it is clear that the potential contribution of DTI to connectivity studies lies not in disproving the existence of postulated connections, but in the unique potential for detecting hitherto unconsidered direct anatomical connections. This is because DTI may be prone to type II errors, but it is far less likely to consistently produce type I errors when connections are averaged across subjects. It is hence highly significant that we have also found evidence for a number of paths which have not been taken into account in the original studies. Incorporating these paths in a new analysis of these models can potentially have a significant impact on the interpretation of these models, since they point to improvements in the anatomical validity of the models, which in turn leads to more veridical path coefficients.

At the current state of technology in effective connectivity, one has considerable freedom to choose the connections in a model and to evaluate different models with different connectivity profiles against each other [35]. This model selection procedure can be augmented significantly with DTI-based tractography, because models in which the connections to a large extent overlap with the connections found in a DTI analysis should in turn be more likely. One could potentially formalize this in a Bayesian framework by designing priors on the connections and by subsequently making the priors on the “known” connections high and sharp and the priors on the “unknown” connections relatively noninformative.

We have observed a large intersubject variability in our findings. If this would be a veridical variability, it would be a surprising and new finding, since the intersubject variability of anatomical connections is generally considered to be low and is furthermore difficult to assess with either DTI or tracer methods. In DTI, the normalization of findings still makes it difficult to compare findings across subjects, whereas tracer studies are normally performed in very few animals because of ethical considerations, which makes any discussion about differences between animals extremely difficult. Although we have not normalized the fiber tracks to a template, we do believe that normalization problems still play an important role in our studies because they might cause the erroneous placement of seed regions in some subjects, which in turn would lead to misleading tractography results and the above-mentioned intersubject variability. The smoothing strategy, employed in functional studies to reduce the effect of anatomical differences between subjects, cannot be applied in our framework because the directional information, used in the tracking procedure, would be smeared out over other voxels and potentially other tracks, with unpredictable implications for the veridicality of the tracks found. Currently, there is no convincing way of solving this problem, as the basic anatomical landmarks vary substantially over subjects.

Recently, advances have been made towards the estimation of multiple fiber directions within one voxel and also in probabilistic tractography. We will now discuss each of these developments and their potential use for our framework.

The estimation of multiple fiber compartments per voxel, in general, brings this technique closer to producing veridical anatomical connections, and a number of techniques have been proposed to achieve this [33, 36–39]. There is, however, one problem which cannot be solved by this technique alone, and that is the kissing/crossing fiber problem: when a fiber has to track through a voxel with multiple compartments, it is uncertain which compartment has to be used to determine the direction in which the track is to be continued. In a number of studies, the direction that is most collinear with the incoming fiber was chosen, but this does not necessarily have to be the true direction. Whereas the single tensor model is probably too conservative in the connections it yields, multiple compartment models might thus yield a number of false positives.

In probabilistic fiber tracking, a measure of uncertainty of the local fiber direction is estimated per voxel [40–44]. Fiber tracking is now done in a Monte Carlo type experiment: the tracking is performed multiple times, each time with a different orientation drawn from the local fiber direction distributions. Subsequently, the number of times a target voxel was hit by this procedure is calculated, which then is converted to an informal measure of “probability of connection”. While this procedure by itself seems valid, in practice this leads to widespread patterns of connectivity and it is uncertain at which level of “probability” the map should be thresholded. Moreover, the probability of connection tends to decrease with increasing distance. In a SEM network, both relatively local and long connections can be included, which makes the comparison of these connections difficult. Thus while both techniques (multiple direction estimation and probabilistic fiber tracking) can potentially alleviate some of the problems, we have encountered (e.g., interhemispheric connections), unresolved issues remain.

In conclusion, we have shown that DTI-based tractography can be used to explore the anatomical connections between regions, used in effective connectivity studies, notwithstanding the current limitations of this method. We have observed evidence for the proposed paths in a large number of cases and, more importantly, we have shown in several cases direct connections that were not included in the original models. We therefore conclude that DTI-based tractography is a valuable tool for exploring the anatomical basis of functional networks.

## Figures and Tables

**Table 1:**
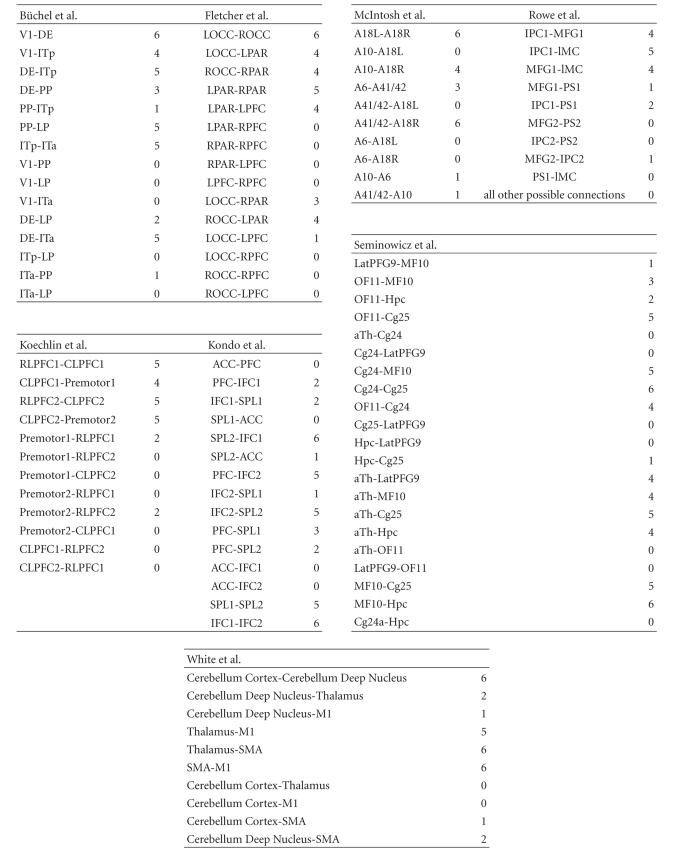
Tractography results for all the proposed networks. The number indicates the number of subjects in which this path was found. Results in black indicate proposed paths, while results in red indicate paths that were not proposed in the original networks.

## APPENDIX

See Table 1.
